# Supplementary material for: Survey on the Mental Health of Dispensing Pharmacists in the Auvergne-Rhône-Alpes Region (France)
Source: Int J Environ Res Public Health. 2023 Oct 28;20(21):6988. doi: 10.3390/ijerph20216988 (PMC10648574; doi:10.3390/ijerph20216988)
Supplement: Supplementary file 1 [file ijerph-20-06988-s001.zip › ijerph-2641157-supplementary.pdf]

\linebreakSupplemental data

Table S1. Qualitative variables of the 3 clusters.

Clusters		1		2		3	
		v.test	*p*	v.test	*p*	v.test	*p*
Professional burnout	Low	18	$<2 \times 10^{-16}$	-8.6	$<2 \times 10^{-16}$	-10	$<2 \times 10^{-16}$
	High	−11	$<2 \times 10^{-16}$	−11	$<2 \times 10^{-16}$	20	$<2 \times 10^{-16}$
	Moderate	−7.8	5.8×10^{-15}	20	$<2 \times 10^{-16}$	−12	$<2 \times 10^{-16}$
Loss of personal accomplishment	Low	6.9	4.4×10^{-12}	ns	ns	−6.7	2.5×10^{-11}
	High	−4.3	1.6×10^{-5}	ns	ns	4.9	1.2×10^{-6}
	Moderate	−4.3	1.5×10^{-5}	ns	ns	3.4	0.00075
Depersonalisation	Low	7.1	1.5×10^{-13}	ns	ns	−7.6	2.4×10^{-16}
	High	−6	2.4×10^{-9}	ns	ns	7	3.6×10^{-12}
HAD Anxiety	Definitive Symptomatology	−9.6	$<2 \times 10^{-16}$	ns	ns	9.9	$<2 \times 10^{-16}$
	No symptomatology	8.8	$<2 \times 10^{-16}$	ns	ns	−9.2	$<2 \times 10^{-16}$
HAD Depression	Definitive symptomatology	−6.8	9.8×10^{-12}	−4.7	2.5×10^{-6}	9.4	$<2 \times 10^{-16}$
	Doubtful symptomatology	−5.4	8.2×10^{-8}	ns	ns	3.7	$<2 \times 10^{-16}$
	No symptomatology	9.4	$<2 \times 10^{-16}$	2.8	0.00500	−10	0.00023
Number of hours worked per week	More than 50 h	−4.4	8.6×10^{-6}	ns	ns	3.1	0.00218
	35 to 50 h	3.8	0.00016	ns	ns	−2.5	0.01233
Lifestyle	Couple	ns	ns	2	0.04722	−2.8	0.00502
	Alone	ns	ns	−2	0.04722	2.8	0.00502
Number of pharmacists in the pharmacy	1	ns	ns	ns	ns	2.1	0.03683

Pharmacy typology	Rural environment	ns	ns	ns	ns	−2.1	0.03948

ns: not significant.

The aim here is to identify clusters of pharmacists with common characteristics based on the selected variables. In these tables, p is the significance of the variable's modality within a cluster, and v-test represents the effect size: the higher the v-test (> 0), the more characteristic the modality is of the cluster; the lower the v-test (< 0), the more absent the modality is from the cluster. When the v-test is close to 0, this means that this modality is neutral; its presence or absence is not characteristic of the cluster.

Table S2. Description of clusters (quantitative variables).

TAG practice in the pharmacy	No	24 (23.1%)	18 (20%)	41 (24.7%)	83 (23.1%)
	Yes	80 (76.9%)	72 (80%)	125 (75.3%)	277 (76.9%)
COVID vaccination practice in the pharmacy	No	7 (6.73%)	4 (4.44 %)	15 (9.04%)	26 (7.22%)
	Yes	97 (93.3%)	86 (95.6%)	151 (91%)	334 (92.8%)
Length of time in the profession (since graduation)	Under 10 years	7(6.73%)	10 (11.11%)	9 (5.42%)	26 (7.22%)
	10 to 20 years	38 (36.5%)	28 (31.1%)	53 (31.9%)	119 (33.06%)
	More than 20 years	59 (56.7%)	52 (57.8%)	104 (62.7%)	215 (59.7%)
Number of hours worked per week	Under 35h	6 (5.77%)	3 (3.33%)	3 (1.81%)	12 (3.33%)
	35 to 50h	73 (70.2%)	45 (50%)	79 (47.6%)	197 (54.7%)
	More than 50h	25 (24%)	42 (46.7%)	84 (50.6%)	151 (41.9%)
Gender	Female	66 (63.5%)	59 (65.6%)	107 (64.5%)	232 (64.4%)
	Male	38 (36.5%)	31 (34.4%)	59 (35.5%)	128 (35.6%)
Age	25 to 40 years	21 (20.2%)	20 (22.2%)	27 (16.3%)	68 (18.89%)
	40 to 55 years	52 (50%)	41 (45.6%)	87 (52.4%)	180 (50%)
	More than 55 years	31 (29.8%)	29 (32.2%)	52 (31.3%)	112 (31.11%)
Lifestyle	Couple	92(88.5%)	82 (91.1%)	131 (78.9%)	305 (84.7%)
	Alone	12 (11.54%)	8 (8.89%)	35 (21.08%)	55 (15.28%)
Presence of children at home	No	31 (29.8%)	32 (35.6%)	47 (28.3%)	110 (30.6%)
	Yes	73 (70.2%)	58 (64.4%)	119 (71.7%)	250 (69.4%)
Pharmacy typology	rural	43 (41.3%)	37 (41.1%)	51 (30.7%)	131 (36.4%)
	semi-urban	32 (30.8%)	28 (31.1%)	62 (3+7.3%)	122 (33.9%)

	urban	29 (27.9%)	25 (27.8%)	53 (31.9%)	107 (29.7%)
Number of customers per day in the pharmacy	0 to 100	22 (21.2%)	10 (11.1%)	24 (14.5%)	56 (15.56%)
	100 to 200	41 (39.4%)	41 (45.6%)	75 (45.2%)	157 (43.6%)
	200 to 400	32 (30.8%)	32(35.6%)	57 (34.3%)	121 (33.6%)
	More than 400	9 (8.65%)	7 (7.78%)	10 (6.02%)	26 (7.22%)
Number of pharmacists in the pharmacy	2 to 3	62 (59.6%)	59 (65.6%)	101 (60.8%)	222 (61.7%)
	4 and more	30 (28.8%)	22 (24.4%)	34 (20.5%)	86 (23.9%)
	1	12 (11.5%)	9 (10%)	31 (18.7%)	52 (14.4%)
Total		104 (28.9%)	90 (25%)	166 (46.1%)	360 (100.0%)

Figures S1 to S3 : Graphic representations of chi² test comparing clusters regarding MBI's dimensions

The bigger and the darker the circle is, the stronger the correlation is. Blue circle denotes a positive correlation between the cluster and the score whereas red denotes a negative correlation.

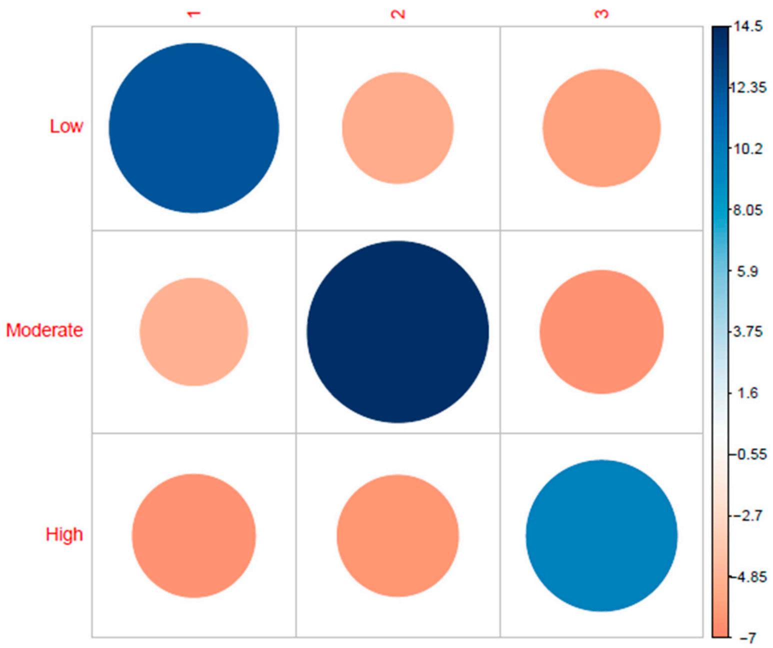

Figure S1. Visualization of the correlation matrix between the cluster (1 to 3) and the Emotional exhaustion score.

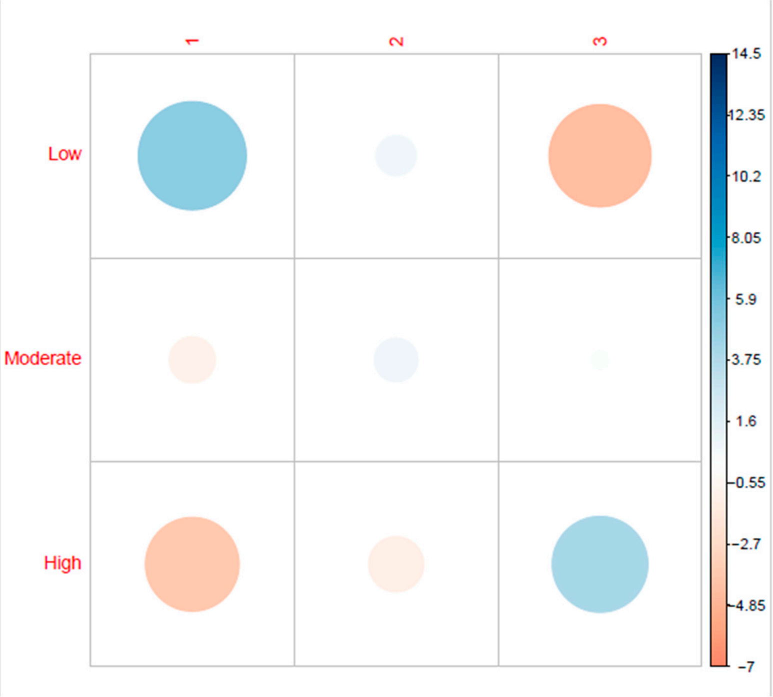

Figure S2. Visualization of the correlation matrix between the cluster (1 to 3) and the Depersonalization score**.**

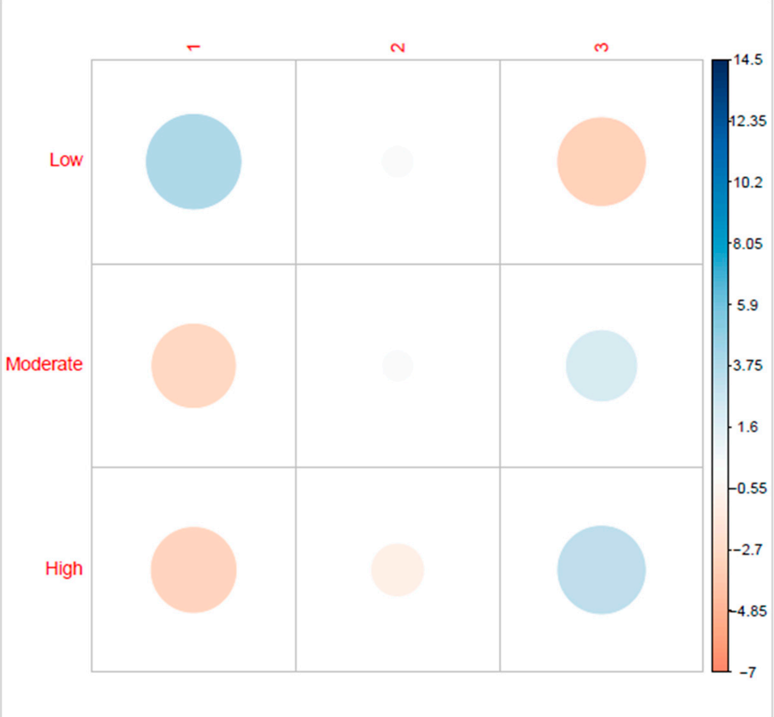

Figure S3. Visualization of the correlation matrix between the cluster (1 to 3) and the Loss of personal accomplishment.
